# Immediate *versus* early urinary catheter removal after gastrectomy under enhanced recovery after surgery protocols: randomized clinical trial

**DOI:** 10.1093/bjsopen/zraf088

**Published:** 2025-08-19

**Authors:** Chen Wei, Gang Wang, Hai-Feng Wang, Hua-Feng Pan, Zhi-Wei Jiang, Mu-Wen Qu

**Affiliations:** Department of Colorectal Surgery, Guang’anmen Hospital, China Academy of Chinese Medical Sciences, Beijing, China; Department of General Surgery, Affiliated Hospital of Nanjing University of Chinese Medicine, Nanjing, China; Department of General Surgery, Affiliated Hospital of Nanjing University of Chinese Medicine, Nanjing, China; Department of General Surgery, Affiliated Hospital of Nanjing University of Chinese Medicine, Nanjing, China; Department of General Surgery, Affiliated Hospital of Nanjing University of Chinese Medicine, Nanjing, China; Department of Colorectal Surgery, Guang’anmen Hospital, China Academy of Chinese Medical Sciences, Beijing, China

**Keywords:** urinary retention

## Abstract

**Background:**

Compliance with enhanced recovery after surgery (ERAS) protocols in gastrectomy, including urinary catheter management, remains poor. This study evaluated the feasibility of immediate urinary catheter removal after radical gastrectomy.

**Methods:**

This was a non-inferiority randomized clinical trial performed at a university-affiliated hospital in China. Patients undergoing radical gastrectomy were randomized in a 1 : 1 ratio to either immediate removal (IR) or early removal (ER) of the urinary catheter. The randomization sequence was computer generated; the investigators and patients were not blinded to group allocation. ERAS protocols were applied in all patients. The primary outcome measure was postoperative urinary retention with a non-inferiority margin of 10% to compare IR with ER. Secondary outcomes were patient comfort, patient anxiety, and depression. Data were analysed using intention-to-treat analysis.

**Results:**

Initially, 248 patients were assessed for eligibility for this study. Data were analysed for 92 patients in the IR group and 89 patients in the ER group. The incidence of postoperative urinary retention was 4.4% and 3.4% in the IR and ER groups, respectively (*P* = 0.733; 1.0% difference, 95% confidence interval −4.6 to 6.6%). Patient comfort levels were significantly higher in IR than ER group (mean(standard deviation) Kolcaba General Comfort Questionnaire score 74.9(7.6) *versus* 72.5(8.0), respectively; *P* = 0.041).

**Conclusion:**

IR of the urinary catheter after gastrectomy is feasible under ERAS perioperative care protocols. It does not increase the incidence of postoperative urinary retention and can provide a more comfortable postoperative experience. Successful IR implementation probably relies on multimodal analgesia and goal-directed fluid therapy.

**Registration number:**

NCT06718114 (http://www.clinicaltrials.gov).

## Teaser

This study was a non-inferiority randomized clinical trial that compared removal of the urinary catheter immediately after gastrectomy to early removal in an enhanced recovery after surgery setting. Immediate catheter removal was found to be feasible. It provided a less stressed postoperative experience while not increasing the postoperative urinary retention rate. The key to ensuring immediate removal may be multimodal analgesia and individualized fluid management.

## Introduction

Urinary catheterization is a routine intervention during radical gastrectomy. Although the procedure has minimal impact on the pelvic organs and nerves in the lower abdomen, consensus on the optimal timing for urinary catheter removal is lacking. Several factors may affect the timing of catheter removal, including a long duration of surgery, significant trauma, prolonged postoperative bed rest, substantial fluid administration, and postoperative pain.

In the literature, reports indicate that urinary catheters are typically retained for 3–5 days after radical gastrectomy^1^. However, this practice is associated with potential adverse events, such as urinary tract infections (UTIs), restricted patient mobility, and prolonged hospital stay. The enhanced recovery after surgery (ERAS) protocol, which optimizes perioperative management, has shortened the duration of catheterization to 1–2 days^2^. Nevertheless, the incidence of postoperative UTI remains positively correlated with the duration of catheter placement. Even if catheters are removed within 24 hours (h) after surgery, approximately 20% of patients will develop a UTI^3^, and 47–90% of patients experience catheter-related bladder discomfort^4^. However, early catheter removal is associated with a higher incidence of postoperative urinary retention (POUR)^5^. The dilemma of balancing POUR against UTI requires further investigation.

Recent evidence has shown that immediate removal of a urinary catheter in the case of colorectal and benign gynaecological surgeries^6,7^ does not increase the rate of catheter reinsertion and can promote early patient mobilization and shorten hospital stay. The effects of the immediate removal of a urinary catheter after radical gastrectomy have not been reported. Thus, the present research compared immediate removal (IR) of a urinary catheter with early removal (ER) after radical gastrectomy to determine whether IR is non-inferior to ER in terms of the incidence of POUR.

## Methods

### Study design

This study was a non-inferiority randomized clinical trial conducted at the Affiliated Hospital of Nanjing University of Chinese Medicine from 10 December 2021 to 20 June 2024. This study followed the CONSORT guidelines. In the IR group, the urinary catheter was removed immediately after surgery, whereas in the ER (control) group, urinary catheter was removed within 24 h after surgery. The full study protocol is provided in the *Supplementary material*.

### Inclusion and exclusion criteria

This trial enrolled adult patients with gastric cancer who were planned for elective laparoscopic radical gastrectomy, with ERAS protocols applied as perioperative management. Patients were excluded if they had a history of benign prostatic hyperplasia or urogenital tumours, stricture, deformities, or surgeries; were taking medication that affects urination (α_1_-adrenoceptor antagonists, anticholinergic drugs, antidepressants, antipsychotics, diuretics, and antihistamines); or preoperative urinalysis indicated a UTI. Patients were considered to have dropped out of the study if they sustained an intraoperative urinary tract injury, experienced life-threatening complications, or withdrew consent.

### Sample size

This trial used the POUR rate as the primary outcome measure. A non-inferiority margin of 10% was determined to be clinically acceptable. Based on a small retrospective review of patients in the Affiliated Hospital of Nanjing University of Chinese Medicine, the POUR rate in the IR group was assumed to be 4.71%, whereas that in the ER group was assumed to be 3.88% based on a literature review^8^. Thus, at least 172 patients (86 patients per group) were required for the study to have 80% power to detect a non-inferiority margin of 10% with a one-sided α of 0.025, allowing a 10% drop-out rate. The sample size was calculated using PASS, version 15.0 (NCSS LLC, Kaysville, Utah, USA).

### Randomization and masking

After study enrolment, patients were randomized 1 : 1 to either the IR group or the ER group. A random permuted block design was used for randomization, and the randomization sequence was computer generated. The investigators and patients were not blinded to treatment allocation.

## Intervention

### Urinary catheter management

**Fig. 1:**
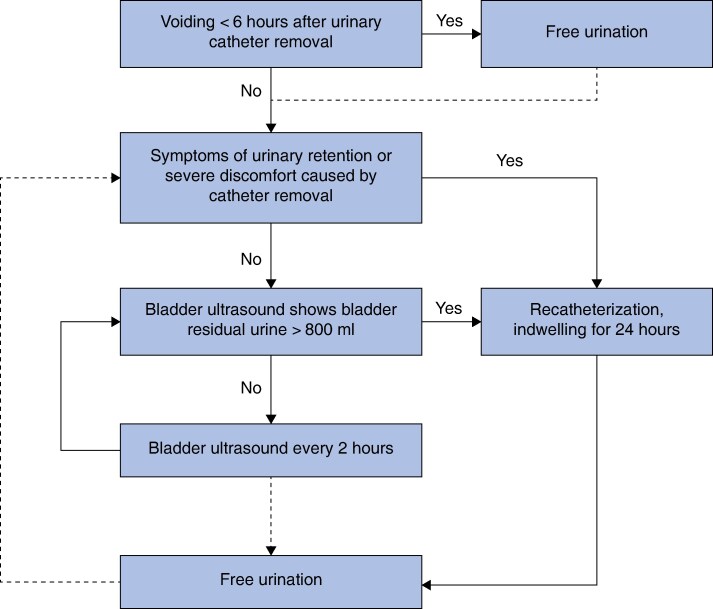
Flow chart of postoperative urinary catheter management Dashed arrows represent the ‘if’ condition.

In all patients, a 14-Fr Foley catheter was placed by a physician after the induction of anaesthesia. The time of catheter removal was according to randomization. Patients in the IR group had their catheter removed in the operating room at the end of the surgery, whereas patients in the ER group had their catheter removed within 24 h after surgery (as recommended by ERAS guidelines). Patients were recatheterized for 24 h if: they were experiencing symptoms of POUR, such as an inability to void, discomfort or pain in the lower abdomen, or severe discomfort after catheter removal; or they were unable to urinate spontaneously within 6 h after removal of the urinary catheter, had no symptoms of POUR, and a bladder ultrasound showed that the residual urine volume in the bladder was > 800 ml. The management pathway is shown in *Fig. 1*.

### Perioperative management

All patients were managed using the ERAS perioperative care protocol. This protocol involves the early removal of various catheters (nasogastric/nasojejunal tubes, abdominal drains), as well as fluid, anaesthesia, analgesia, and mobilization management.

The aim of fluid management was to achieve a near-zero fluid balance perioperatively. There was no mechanical bowel preparation in any of the patients. Patients were provided with carbohydrate-rich drinks before surgery. Goal-directed fluid therapy was adopted during surgery, guided by pulse contour cardiac output, which was used to continuously monitor haemodynamic parameters. After surgery, patients were encouraged to start early oral feeding instead of parenteral nutrition. Fluid therapy was also guided by clinical assessments and results of haemodynamic monitoring.

All patients were anaesthetized using sevoflurane inhalation combined with intravenous propofol and remifentanil. An epidural block was not performed. Analgesia included infiltration of 0.5% ropivacaine (10–20 ml in total) into the incision before closure, oral administration of hydrocodone hydrochloride, and the intravenous infusion of flurbiprofen ester and methylprednisolone after surgery. Patients received transnasal butorphanol tartrate as needed. Anaesthetists did not perform a transversus abdominis plane block or a rectus sheath block.

Finally, all patients were encouraged to mobilize as soon as possible after the surgery and were instructed to meet daily activity targets.

### Primary outcome

The primary outcome measure in this study was the rate of POUR during hospitalization. POUR was defined as the inability to void completely within 6 h after catheter removal. POUR was diagnosed if a patient had symptoms of urinary retention (for example, an inability to urinate, urinary urgency, a feeling of incomplete bladder emptying, abdominal distension, abdominal pain) or a bladder ultrasound showed the retention of > 800 ml urine^9^.

### Secondary outcomes

Secondary outcome measures were the rate of UTI during hospitalization, patient comfort levels, and the anxiety and depression status. UTI was diagnosed on the basis of the presence of symptoms such as low back pain, pelvic pain, suprapubic tenderness, acute haematuria, and fever. If the urinary catheter had been removed, UTI symptoms may include frequent urination, urgency, and dysuria. Abnormal urinalysis included increased red or white blood cell counts. Patient comfort levels were assessed using the Kolcaba General Comfort Questionnaire (GCQ)^10^, with higher scores indicating greater patient comfort. The Hospital Anxiety and Depression Scale (HADS) was used to evaluate levels of patient anxiety and depression, with scores ≥ 11 indicating an anxious status^11^. Patient comfort levels, anxiety, and depression were assessed at 09.00 hours on the first postoperative day (POD).

### Additional outcomes

Additional outcomes included patient baseline characteristics (sex, age, American Society of Anesthesiologists grade, body mass index, tumour node metastasis (TNM) staging), surgical characteristics (surgery type, surgical approach, operative time, estimated blood loss), and recovery (length of hospital stay after surgery, postoperative complications (except POUR/UTI), time until flatus after surgery, time until ambulation after surgery). Analgesia was assessed using a visual analogue scale (VAS) for pain. Details of intraoperative and postoperative opioid consumption (calculated as morphine milligram equivalent (MME)) on POD 0, POD 1, and POD 2 were recorded. Opioid consumption on POD 0 was calculated from when the patient returned to the general surgery ward or the intensive care unit up until 23.59 hours. Fluid management outcome measures were also recorded, including intraoperative urine volume and intraoperative and postoperative intravenous fluid volume.

### Statistical analysis

Data were analysed using intention-to-treat (ITT) analysis. A non-inferiority analysis was used for POUR. IR was non-inferior to ER if the upper bound of the two-sided 95% confidence interval (c.i.) for the difference in POUR was lower than the predefined margin of 10%. Categorical variables are presented as numbers and percentages, and were compared using the χ^2^ test, Fisher’s exact test, or the Wilcoxon rank-sum test, as appropriate. Continuous variables are presented as the mean with standard deviation (s.d.) or as the median with interquartile range (i.q.r.), and were analysed using *t* tests or the Mann–Whitney *U* test, as appropriate. Analyses were performed using SPSS^®^ version 26 (IBM, Armonk, NY, USA). All statistical tests were two-sided and *P* < 0.050 was considered significant.

### Ethics and trial registration

The trial protocol was approved by the Ethics Committee of the Affiliated Hospital of Nanjing University of Chinese Medicine (2021NL-167-02). The trial was registered with ClinicalTrials.gov (NCT06718114). Written consent was obtained from all participants before enrolment.

## Results

### Study population

**Fig. 2:**
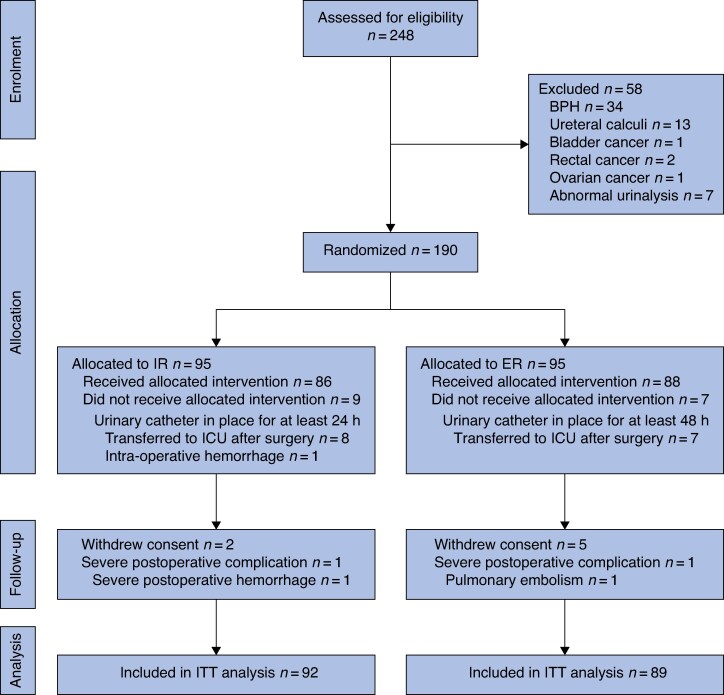
CONSORT flow diagram of the study process BPH, benign prostatic hyperplasia; IR, immediate removal of the urinary catheter; ER, early removal of the urinary catheter; ICU, intensive care unit; ITT, intention-to-treat.

Initially, 248 patients were enrolled in this study. Of these patients, 58 were excluded and 9 were considered to have dropped out because of severe postoperative complications or withdrawal of consent (more detailed information is provided in *Fig. 2*). Thus, there were 92 and 89 patients included in the IR and ER groups, respectively. There were no statistically significant differences between the two groups in age, sex, body mass index, American Society of Anesthesiologists grade, operative time, estimated blood loss, surgical approach, surgical type, or TNM stage (*Table 1*).

**Table 1:** Patient characteristics and surgical outcomes in the IR and ER groups

	IR group (*n* = 92)	ER group (*n* = 89)	P*
**Sex**			
Male	67 (72.8%)	71 (79.8%)	0.272
Female	25 (27.1%)	18 (20.2%)	
Age (years), median (i.q.r.)	65 (65.3–69.0)	65 (58.5–70.5)	0.170
BMI (kg/m^2^), median (i.q.r.)	23.1 (21.5–25.1)	24.1 (22.3–25.0)	0.202
ASA grade III or IV	12 (13.0%)	20 (22.5%)	0.096
**Type of surgery**			0.981
Proximal gastrectomy	26 (28.3%)	27 (30.3%)	
Distal gastrectomy	44 (47.8%)	39 (43.8%)	
Total gastrectomy	22 (23.9%)	23 (25.8%)	
**Surgical approach**			0.852
Open	1 (1.1%)	2 (2.2%)	
Laparoscopic	39 (42.4%)	35 (39.3%)	
Robotic	52 (56.5%)	52 (58.4%)	
Operative time (min), median (i.q.r.)	232.5 (196.3–280.0)	240.0 (215.0–272.5)	0.487
Estimated blood loss (ml), median (i.q.r.)	55.0 (50.3–64.8)	56.0 (50.5–67.0)	0.588
**TNM classification**			0.679
Stage I	37 (40.2%)	38 (42.7%)	
Stage II	29 (31.5%)	27 (30.3%)	
Stage III	20 (21.7%)	21 (23.6%)	
Stage IV	6 (6.5%)	3 (3.4%)	

Values are *n* (%) unless otherwise stated. *Age, BMI, operative time, estimated blood loss were analysed using Mann–Whitney *U* test. Sex was compared using the χ2 test. Type of surgery, surgical approach, TNM classification were compared using the Wilcoxon rank-sum test. IR, immediate removal of the urinary catheter; ER, early removal of the urinary catheter; i.q.r., interquartile range; BMI, body mass index; ASA, American Society of Anesthesiologists; min, minutes; TNM, tumour node metastasis staging system of the American Joint Committee on Cancer (8th edition).

### Primary outcome

POUR was diagnosed in 7 of 181 patients, for a total POUR incidence of 3.9%. Four patients (4.4%) in the IR group and 3 (3.4%) in the ER group experienced POUR (*P* = 0.733). The difference in the POUR rate between the two groups was 1.0% (95% c.i. −4.6% to 6.6%). Because the upper bound of the 95% c.i. was lower than the non-inferiority margin of 10% and the 95% c.i. contained 0, the IR of the urinary catheter after radical gastrectomy is non-inferior to ER. All patients with POUR were recatheterized, with five catheters removed after 24 h and two removed after 48 h without further urinary retention (*Table 2*). Ten patients reported discomfort or pain while urinating, haematuria was recorded in three patients, and six patients reported urgent urination after catheter removal; none of these patients were recatheterized, and all symptoms resolved within 24 h. No severe complications related to the urinary catheter were reported.

**Table 2:** Primary and secondary outcomes for the IR and ER groups

	IR group (*n* = 92)	ER group (*n* = 89)	Difference*	*P*†
POUR	4 (4.4%)	3 (3.4%)	1.0 (−4.6, 6.6)	0.733
UTI	0 (0%)	4 (4.5%)	−4.5 (−8.8, −0.3)	0.121
Increased anxiety/depression‡	25 (27.2%)	53 (59.6%)	−32.4 (−46.0, −18.7)	<0.001
Kolcaba GCQ score, mean(s.d.)	74.9(7.6)	72.5(8.0)	2.4 (0.1, 4.7)	0.041

Values are *n* (%) unless otherwise stated. *Values in parentheses are 95% confidence intervals. ‡Measured using the Hospital Anxiety and Depression Scale (HADS), with scores > 8 considered to indicate anxiety or depression. †POUR and UTI were compared using Fisher’s exact test. Increased anxiety/depression were compared using the χ2 test. Kolcaba GCQ score was analysed using *t* tests. IR, immediate removal of the urinary catheter; ER, early removal of the urinary catheter; POUR, postoperative urinary retention; UTI, urinary tract infection; GCQ, general comfort questionnaire; s.d., standard deviation.

### Secondary outcomes

There was no significant difference in the UTI rate between the IR and ER groups (0 *versus* 4.5%, respectively; *P* = 0.121; *Table 2*). According to HADS scores, 25 (27.2%) and 53 (59.6%) patients had increased anxiety in the IR and ER groups, respectively (*P* < 0.001; *Table 2*). The mean(s.d.) Kolcaba GCQ score was 74.9(7.6) in the IR group and 72.5(8.0) in the ER group (*P* = 0.041; *Table 2*).

### Additional outcomes

Specific data on additional outcome measures are presented in *Table 3*. There were no significant differences in intraoperative and postoperative intravenous fluid volume from POD 0 to POD 2 between the IR and ER groups. All patients received effective analgesia, with a VAS score for pain ≤ 3 from POD 0 to POD 2. There was also no significant difference between the IR and ER groups in intraoperative and postoperative opioid consumption. The median time until ambulation in the IR and ER groups was 15 (i.q.r. 10.3–21.8) and 21 (i.q.r. 17.0–25.0) h, respectively (*P* < 0.001). The median postoperative length of hospital stay was 6 (i.q.r. 5.0–7.0) and 6 (i.q.r. 5.0–7.5) days in the IR and ER groups, respectively (*P* = 0.047). There were no significant differences in time until flatus and the incidence of postoperative complications between the two groups.

**Table 3:** Additional outcomes for the IR and ER groups

	IR group (*n* = 92)	ER group (*n* = 89)	P*
Intraoperative i.v. fluid (ml), median (i.q.r.)	1825.5 (1543.3–2738.0)	2083.0 (1562.5–2568.0)	0.434
Intraoperative urine (ml), median (i.q.r.)	447.5 (263.5–749.5)	459.0 (308.5–834.5)	0.223
Postoperative i.v. fluid (ml), median (i.q.r.)	5950.0 (4662.5–7537.5)	6400.0 (5350.0–7900.0)	0.060
**VAS for pain**			
POD 0, median (i.q.r.)	3 (3–4)	3 (3–4)	0.172
POD 1, median (i.q.r.)	2 (2–3)	2 (2–2)	0.199
POD 2, median (i.q.r.)	2 (2–2)	2 (2–2)	0.481
**Opioid consumption**			
Intraoperative MME (mg), median (i.q.r.)	28.32 (25.5–30.5)	27.9 (25.8–29.1)	0.111
POD 0 MME (mg), median (i.q.r.)	15.0 (15.0–20.0)	15.0 (15.0–20.0)	0.767
POD 1 MME (mg), median (i.q.r.)	30.0 (30.0–35.0)	30.0 (30.0–35.0)	0.362
POD 2 MME (mg), median (i.q.r.)	30.0 (30.0–38.8)	30.0 (30.0–40.0)	0.550
Time until flatus (h), median (i.q.r.)	31.8 (28.2–40.9)	31.5 (26.9–40.4)	0.806
Time until ambulation (h), median (i.q.r.)	15.0 (10.3–21.8)	21.0 (17.0–25.0)	<0.001
Postoperative complications	10 (10.9%)	10 (11.2%)	0.937
LOS after surgery (days), median (i.q.r.)	6.0 (5.0–7.0)	6.0 (5.0–7.5)	0.047

Values are *n* (%) unless otherwise stated. *Postoperative complications were compared using the χ2 test. Other variables were analysed using Mann–Whitney *U* test. IR, immediate removal of the urinary catheter; ER, early removal of the urinary catheter; i.v., intravenous; i.q.r., interquartile range; VAS, visual analogue scale; POD, postoperative day; MME, morphine milligram equivalent; h, hours; LOS, length of hospital stay.

## Discussion

This randomized clinical trial (RCT) found that removing urinary catheter immediately after radical gastrectomy was non-inferior to ER when applying ERAS strategies. Therefore, removing the catheter immediately may help create a more comfortable postoperative patient experience.

ER of the urinary catheter can address many of the adverse effects associated with urinary catheterization, including catheter-related agitation, UTI, and prolonged hospital stay. POUR is the main concern regarding early catheter removal, with an incidence ranging from 5 to 70%^16^. Using the ERAS protocol, the incidence of POUR has been reported to range from 0 to 13.17% when catheters are removed within 48 h after gastrectomy^8^. Immediate postoperative urinary catheter removal remains controversial^17–19^. Althoff *et al*.^20^ retrospectively analysed the incidence of acute urinary retention in patients who underwent colorectal surgery with either no urinary catheterization, immediate catheter removal after surgery, catheter removal within 24 h of surgery, or catheter removal > 24 h after surgery and found no significant differences among the groups. In that study, the incidence of urinary retention in the IR group was 6.8%^19^. The opposite result was reported in a retrospective study^21^, in which the POUR rate in the group with immediate urinal catheter removal after laparoscopic Nissen fundoplication was 24.4%, 1.5-fold higher than in the groups in which the catheter was removed on the night of the surgery or on POD 1. Although radical gastrectomy for gastric cancer is associated with significant trauma, a long operative time, and complex stress, in the present study the incidence of POUR in the IR group was 4.4%, which is lower than reported in previous studies and non-inferior to the POUR rate in the ER group. This may be attributed to the use of the ERAS perioperative management protocol in this study, in which multimodal analgesia and personalized perioperative fluid management play significant roles.

The occurrence of POUR is also related to opioid consumption during the perioperative period^22^. Opioids bind to both μ- and δ-opioid receptors in the spinal cord. This reduces the discharge of parasympathetic nerve fibres in the sacral cord, leading to urinary retention. To achieve effective analgesia while sparing the use of opioids, the ERAS guidelines recommend a multimodal analgesia regimen after laparoscopic surgery, namely a combination of the infiltration of a local anaesthetic into the wound, low-dose opioid patient-controlled intravenous analgesia (PCIA), and the use of non-steroidal anti-inflammatory drugs. Lanz *et al*.^23^ found that the incidence of POUR is higher with PCIA than following the intramuscular administration of morphine or pethidine. This indicates that the prolonged duration of stable effective plasma concentrations with PCIA has an extended adverse effect on bladder function. Therefore, in this study ERAS recommendations were combined with PROSPECT guidelines^24^: PCIA was replaced with the oral κ-opioid receptor agonist oxycodone hydrochloride and the use of methylprednisolone and ropivacaine as foundational analgesics after surgery was expanded. This regimen addresses incision, visceral, and inflammatory pain through multiple routes of administration, targeting peripheral and central analgesic pathways. The median postoperative MME in this study was 30 mg/day, which achieved comparable analgesic effects as reported in other gastric cancer-related studies (with a median postoperative VAS score of ≤ 3)^25^ and was lower than that in the traditional analgesic regimen required after radical gastrectomy (typically 37.5 mg/day MME^26^).

POUR is also associated with perioperative fluid management. A retrospective study^27^ found that the risk of developing POUR is significantly increased when the volume of intravenous infusion is greater than 1350 ml for total joint arthroplasty patients (odds ratio 2.88; 95% c.i. 1.25 to 6.59; *P* = 0.013). The intravenous infusion of large amounts of fluid will cause rapid filling of the bladder. When bladder capacity exceeds maximum capacity for 2–3 h, the bladder detrusor may be injured, causing urinary retention. In consideration of postoperative gastrointestinal dysfunction and preventing anastomotic fistula, intravenous infusion of 2000–3000 ml/day is required due to long-term fasting after gastrectomy. In this study, current guidelines were following, such as the intake of clear fluids up to 2 h and solid food up to 6 h before the induction of anaesthesia, no mechanical bowel preparation, goal-directed fluid therapy, and postoperative oral feeding as early as possible. These strategies resulted in a smaller perioperative intravenous infusion volume (approximately 500–2000 ml depending on a patient’s condition). This is in line with the findings of Meillat *et al.*^28^, who reported that a goal-directed intraoperative fluid infusion protocol of <3 ml per kg per h) can significantly improve the success rate of catheter removal. Furthermore, a lower volume of intravenous infusion will have less of an effect on haemodynamic stability, which means that monitoring urine output to guide fluid infusion is not as important in the current as in earlier times. However, the relationship between POUR and fluid administration remains inconsistent. For example, in a prospective study, Scholten *et al*.^29^ determined the incidence of and risk factors for POUR after lower limb joint replacement guided by ERAS and found no causal relationship between perioperative fluid therapy and POUR. Unfortunately, that study did not differentiate between fluid intake methods (oral or intravenous); nor did it specify infusion volume^29^, which indicates that the results may not be comparable to those of the present study. Brouwer *et al.*^30^ reached a similar conclusion in their study of patients undergoing general surgery who did not require intraoperative catheterization. This may be related to the fact that the surgery in that study did not involve fasting and extensive intravenous fluid therapy^30^.

In terms of assessing patient comfort, Kolcaba GCQ^10^ scores differed significantly between the IR and ER groups, but the mean difference was only 2.4 (95% c.i. 0.1 to 4.7; *P* = 0.041). Because both groups are considered to be early removal groups, this difference may not be clinically significant. The median postoperative hospital stay for both groups was 6 days, with no significant difference. However, a previous study^31^ indicated that the presence of a prolonged indwelling catheter can extend the length of hospital stay. This apparent discrepancy is likely due to the fact that both groups in the present study were managed using the ERAS perioperative care protocol, and there was no traditional catheter removal group. In the present study, patients in the IR group mobilized earlier after surgery than patients in the ER group, which is consistent with previous literature^32^.

This study has some limitations. First, it was a single-centre study with a homogeneous participant population, all of whom were Chinese and most of whom were from south-east coastal cities of mainland China. There may be selection bias, although there is reports that race or ethnicity is related to POUR. The present study was not masked due to the nature of intervention and data on patients’ preoperative anxiety and depression status were not collected. A significantly higher proportion of patients in the ER group were found to have anxiety or depression after surgery than in the IR group. Although the placement of an indwelling catheter is a minor intervention, patients may assume that their condition is too severe for them to urinate independently, resulting in unnecessary anxiety. There is evidence that patients experience shame and helplessness due to the presence of a urinary catheter, which can lead to anxiety^12^. Taking these findings into consideration, removal of the urinary catheter in the operating room may result in a less anxious postoperative experience for patients; however, the results in this study regarding anxiety/depression after surgery in the IR and ER groups (27.2% *versus* 59.6%, respectively) could be exaggerated because of information bias.

The prophylactic use of antibiotics has been reported to significantly reduce the incidence of UTI^13^. In the present study, all patients underwent radical gastrectomy for gastric cancer and required prophylactic antibiotics, and this may have masked differences in the incidence of UTIs between the IR and ER groups. In consideration of cost savings, in the present study bladder ultrasound was only performed in patients who did not spontaneously void within 6 h after surgery, rather than in all patients. Therefore, the POUR rate we report may be lower than the actual rate because of missing asymptomatic patients with a residual urine volume >800 ml within 6 h after surgery. However, it has been argued that the diagnosis of POUR should be symptom-oriented rather than based on laboratory indicators^14^.

This is the first RCT to examine the effects of urinary catheter removal immediately after radical gastrectomy within an ERAS protocol. China has one of the highest rates of advanced-stage gastric cancer globally, along with rates of radical gastrectomy; performing such a trial in other countries with a lower incidence of gastric cancer may be challenging^15^.

In conclusion, immediate urinary catheter removal after radical gastrectomy is feasible when applying ERAS protocols. Immediate urinary catheter removal provides a less stressed postoperative experience while not increasing the rate of POUR. To ensure immediate urinary catheter removal, a combination of various elements of the ERAS perioperative care protocol is needed, especially multimodal analgesia and goal-directed fluid management.

## Supplementary Material

zraf088_Supplementary_Data

## Data Availability

Data are available from the corresponding authors upon reasonable request.
