# Supplementary material for: Immediate versus early urinary catheter removal after gastrectomy under enhanced recovery after surgery protocols: randomized clinical trial
Source: BJS Open. 2025 Aug 19;9(4):zraf088. doi: 10.1093/bjsopen/zraf088 (PMC12361889; doi:10.1093/bjsopen/zraf088)
Supplement: zraf088_Supplementary_Data [file zraf088_supplementary_data.docx]

**Immediate versus early urinary catheter removal after gastrectomy under enhanced recovery after surgery protocols: randomized controlled trial**

Authors Chen Wei^1^, Gang Wang^2^, Hai-Feng Wang^2^, Hua-Feng Pan^2^, Zhi-Wei Jiang^2,*^and Mou-Wen Qu^1,*^

^1^Department of Colorectal Surgery, Guang’anmen Hospital, China Academy of Chinese Medical Sciences, Beijing, China

^2^Affiliation Department of General Surgery, Affiliated Hospital of Nanjing University of Chinese Medicine, Nanjing, China

**Corresponding author.** Zhi-Wei Jiang, Department of General Surgery, Affiliated Hospital of Nanjing University of Chinese Medicine, No.155 Hanzhong Road, Nanjing 210029, China;

Tel: +86-025-86617141; e-mail: surgery34@163.com; **ORCID ID** 0009-0009-2316-3850

Mou-Wen Qu, Department of Colorectal Surgery, Guang’anmen Hospital, China Academy of Chinese Medical Sciences, No.5 North Line Pavilion, Beijing 100053, China

Tel: +86-010-88001025; e-mail: gamyygck@163.com; **ORCID ID** 0009-0000-0471-3997

**Supplementary Materials - Index**

| **Supplementary Methods** |  |
| --- | --- |
| Study Protocol in Englisch | *page 2-5* |
| Study Protocol in Chinese |  |

**Supplementary Methods**

**Immediate versus Early Urinary Catheter Removal After Gastrectomy under Enhanced Recovery After Surgery Protocols: A Randomized Controlled Trial Research Protocol**

**Trial registration:** ClinicalTrials.gov (NCT06718114)

**Protocol version:** 2.0

**Sponsor:** Affiliated Hospital of Nanjing University of Chinese Medicine

**Collaborators:** Guang'anmen Hospital of China Academy of Chinese Medical Sciences

**Correspondence:** Jiang Zhiwei

**E-mail:** surgery34@163.com;

**Funding:** None.

**Introduction**

Background: Colorectal and benign gynecological surgeries has realized removal of urinary catheter in the operating room, while gastrectomy hasn't been explored for such possibility. Immediate removal of urinary catheter may cause urinary retention, whereas catheter implementation is related to urinary tract infection, catheter-related discomfort, prolonged hospital stay.

Objective: This study aims to evaluate the feasibility of immediate urinary catheter removal after radical gastrectomy.

Trial design: This is a noninferiority, randomized Controlled Trial, with 2 parallel groups. Participants will be allocated to each group at a 1:1 ratio. Arms and Interventions are as follow:

| Arm | Intervention |
| --- | --- |
| Experimental: immediate removal of urinary catheter | Procedure: The urinary catheter will be removed immediately after surgery, in the operating room. |
| Early removal of urinary catheter | Procedure: The urinary catheter will be kept after surgery and be removed within 24 hours after surgery. |

**Methods**

**Study setting**: Affiliated Hospital of Nanjing University of Chinese Medicine, a university affiliated Tertiary A hospital in China.

**Eligibility criteria**: This trial enrolled adult patients with gastric cancer planned for elective radical gastrectomy, applying ERAS protocols as perioperative management. Patients were excluded if they had history of benign prostatic hyperplasia, history of urinary system tumors, stricture, deformities, or surgeries, medication intake that affects micturition, or preoperative urinalysis indicated UTI. Patients were considered as dropouts if they had intraoperative urinary tract injury, life-threatening complications, or withdrew consensus.

**Interventions**: All patients will receive ERAS perioperative care. Patients in the immediate removal group will have their catheter removed in the operating room at the end of the surgery by surgeons, while patients in the early removal group will have their catheter removed by surgeons within 24 hours after surgery. If there are postoperative urinary retention symptoms after the removal, a re-catheterization will be done. If the patient is unable to urinate spontaneously within 6 hours after removal and no symptoms of POUR, a bladder ultrasound will be performed. If the bladder ultrasound suggests postoperative urinary retention, which is over 800mL residential urine volume, a re-catheterization will be done. If the bladder ultrasound shows residential urine volume is less than 800mL and the patient cannot urinate, the doctors will consider other possibilities such as not enough volume intake.

**Outcomes**:

**Primary outcome measure**: Postoperative rate during hospitalization.

**Secondary Outcome Measures**:

a. postoperative urinary tract infection during hospitalization

b. comfort status: evaluate at postoperative day 1, measured by Kolcaba scale, Kolcaba scale scores from 30 to 112, higher scores mean the patients feel more comfortable during hospitalization (a better outcome).

c. anxious status: evaluate at postoperative day 1, measured by hospital anxiety and depression scale (HAD scale), HAD scale scores from 0 to 20, a higher score means the patients feel more stressful during hospitalization (a worse result).

**Fluid management outcomes**: Intra-operative intravenous fluid/mL. postoperative intravenous fluid/mL. Intra-operative urine volume/mL.

**Analgesia outcomes**: VAS on POD 0, 1, 2. Opioids consumption was calculated as morphine milligram equivalent/mg, intra-operative, POD 0, 1, 2 will be recorded.

**Recovery outcomes**: Hours until flatus/hours. Hours until ambulation/hours. Postoperative complication, n(%). Duration of hospitalization after surgery/days.

**Patient characteristics**: Gender (male or female), n(%). Age/year. BMI/kg/m2. ASA I, II, III or IV, n(%)

**Surgical outcomes**: Type of surgery, n(%), proportion of proximal gastrectomy, distal gastrectomy, total gastrectomy. Surgical approach, n(%), proportion of open, laparoscopic, robotic surgery. Operative time/minutes. Estimated intraoperative blood loss/mL. TNM stage(I, II, III, IV) n(%).

**Participant time line**

**Sample size**: This study used postoperative urinary retention rate as the primary outcome measure. A noninferiority margin of 10% was determined to be clinically acceptable. we used Chow’s method to calculate sample size. Assuming that the POUR rate in immediate group were 4.71% in IR group based on a small retrospective review before this study carried out and 3.88% in ER group based on literature review. At least a total of 172 patients (86 patients per group) were required to provide 80% power to detect a noninferiority margin of 10% with a 1-sided α of 0.025, allowing a 10% drop out rate.

**Randomization**: After enrollment, patients were randomized to either IR group or ER group at a 1:1 ratio. The randomization sequence was computer-generated, using a permuted block of variable size design (2,4,6). Information on intervention allocation was sent to the surgeon at the end of surgery. Randomization was provided by a research assistant.

**Masking**: The investigators and patients were not blinded to the treatment allocation.

**Data collection, management and analysis**

Data collection and management methods: Each participants’ data will be recorded by a CRF. Then the data will be collected in Excel. Range checks for data value will be done.

Statistical methods: Data analyses were conducted by the intention-to-treat (ITT) protocol. Categorical variables were presented as numbers (percentages) and compared using the χ2 test or Fisher exact test or Wilcoxon rank-sum test, as appropriate. Continuous variables were presented as mean (standard deviation) or median (interquartile range) and analyzed using the t test or Mann-Whitney U test, as appropriate. The analyses were performed using SPSS, version 26 (IBM Corp.). All statistical tests were 2-sided. P value of < 0.05 was considered significant.

**Monitoring**

Data monitoring: None. This study does not have significant safety concerns. We think the benefits of a DMC may not justify the cost. The data will be monitored by the study team periodically.

Harms: Urinary retention, see Intervention section. Other harms may include catheter-associated urinary tract infections, urinary tract trauma, bladder spasm, inability to remove catheter. Catheter assessment will be done at least every 4 hours. If any of the adversities occur, it will be recorded in the CRF and be treated symptomatically. The catheter will be removed as needed.

**Ethics and dissemination**

The trial protocol was approved by the ethics committee of Affiliated Hospital of Nanjing University of Chinese Medicine (2021NL-167-02). The surgeons will talk to the potential trial participants. Written consent will be obtained from all participants before enrollment. Information such as name, date of birth, contact details, medical history, and other relevant data will be collected during the initial screening and enrollment process. All personal information will be stored in secure electronic databases and physical files with restricted access. Access to personal information will be limited to authorized personnel only. Each individual with access will be required to adhere to strict confidentiality protocols. Personal information will be retained for the duration of the trial and for a period afterward as required by regulatory guidelines. After this period, all personal information will be securely destroyed or permanently deleted in accordance with applicable laws and regulations.

There are no financial and other competing interests for principal investigators for the overall trial and each study site.

**Informed consent materials**

Model consent form is originally in Chinese. English translation version will be available on request to corresponding author of the study.

胃癌术后立即拔除和早期拔除导尿管的随机对照研究临床研究方案

组长单位：南京中医药大学附属医院

项目负责人：江志伟

承担科室：普通外科

联系电话：15951953206

参加单位：中国中医科学院广安门医院

研究年限：2021年10月－ 2024年10月

版本号：2.0

版本日期：2021年10月6日

方 案 摘 要

| 项目名称 | 胃癌术后立即拔除和早期拔除导尿管的随机对照研究 |
| --- | --- |
| 研究目的 | 探究胃癌术后立即拔除导尿管的安全性和可行性 |
| 研究设计 | 随机对照试验 |
| 病例总数 | 约172人 |
| 病例选择 | 入选标准 1.病理证实胃癌； 2.拟行胃癌根治术，围术期采用加速康复外科理念；  3.年龄大于等于18岁。 |
|  | 排除标准  1.患者有良性前列腺增生病史、泌尿系统肿瘤病史、狭窄、畸形或手术史；  2.使用影响排尿的药物（α-1受体阻滞剂、抗胆碱能药物、抗抑郁药、抗精神病药、利尿剂和抗组胺药）；  3.术前尿常规检查提示尿路感染。 |
| 治疗方案 | 术后立即拔除导尿管组在手术结束后，由手术医师在手术室拔除导尿管；早期拔除组在术后24小时内由主治医师拔除导尿管。 |
| 疗效评定 | 主要结局指标为术后尿潴留发生率；  次要结局指标为术后尿路感染发生率、患者舒适度评价（由Kolcaba舒适度量表评价）、患者心理评价（由HADS量表评价）。 |
| 统计方法 | 数据分析采用意向性分析（Intention-to-treat, ITT）方案。对于术后尿潴留（Postoperative Urinary Retention, POUR），采用非劣效性分析。如果立即拔除导尿管与早期拔除导尿管的POUR差异的双侧95%置信区间上限低于预先设定的10%的非劣效性界值，则认为立即拔除导尿管不劣于早期拔除导尿管。  分类变量以例数（百分比）表示，采用卡方检验（χ² test）或Fisher精确检验或Wilcoxon秩和检验进行比较，具体根据数据的分布情况而定。连续变量以均值（标准差）或中位数（四分位间距）表示，采用t检验或Mann-Whitney U检验进行分析，具体根据数据的正态性而定。  所有统计分析均使用SPSS 26版（IBM公司）完成。所有统计检验均为双侧检验，P值小于0.05被认为具有统计学意义。 |
| 研究期限 | 2021年10月－ 2024年10月 |

一、研究背景

胃癌根治术中常规留置尿管，虽然手术对下腹部盆腔脏器、神经影响较小，但由于手术时间长，创伤大，患者术后卧床时间长、大量补液、疼痛等原因，术后导尿管最佳拔除时间仍未有共识。

既往文献显示，胃癌根治术需留置导尿管3～5天，但存在尿路感染（Urinary Tract Infection, UTI）、限制患者行动、延长住院时间等不良影响。加速康复外科（Enhance Recovery After Surgery，ERAS）理念通过优化围术期管理措施，将此过程缩短至1~2天。尽管如此，术后UTI与导尿管留置时间正相关，即使术后24h内拔除尿管，仍有20%的患者发生UTI，且高达47%~90%的患者存在导尿管相关膀胱不适。然而，早期拔除导尿管与高术后尿潴留（Postoperative Urinary Retention, POUR）发生率相关，如何解决POUR和UTI的两难关系仍需进一步研究。最近一篇系统综述指出，对于子宫切除术、剖宫产等妇科手术，立即拔除导尿管不会增加导尿管再置入率，还有利于患者早期活动和缩短住院时间。

目前尚无胃癌根治术后立即拔除导尿管的研究。本研究为填补此空白，通过对比胃癌根治术后立即拔除导尿管和早期拔除导尿管，探讨胃癌根治术后立即拔除导尿管的可行性，尤其是在尿潴留发生率方面，立即拔除是否非劣效于早期拔除。

二、研究目的

通过对比胃癌根治术后立即拔除导尿管和早期拔除导尿管，探讨胃癌根治术后立即拔除导尿管的可行性，尤其是在尿潴留发生率方面，立即拔除是否非劣效于早期拔除。

三、研究设计类型、原则与试验步骤

1.研究设计类型：随机对照试验

2.随机化：采用非固定区组的区组随机化，随机化由试验助手提供，随机序列由计算机生成，区组长度随机为4、6、8，分配比例为1:1。试验助手在手术结束时告知手术者该患者的分组为IR组或ER组。

3.盲法：本研究由于干预特性未对研究者及受试者设盲。

4.研究中心：南京中医药大学附属医院，中国中医科学院广安门医院

5.样本量：研究对象的术后急性尿潴留（POUR）发生率为观测的主要评价指标，根据查阅文献及预实验结果，试验组IR组POUR发生率为0.0471（5/106，回顾性数据），ER发生率为0.0388，设单侧α=0.025，把握度即1-β为0.8，试验组与对照组样本量比值为1:1，非劣效界值为10%，参照Chow等方法，采用R语言计算得到试验组样本量为77例，对照组样本量为77例。考虑10%失访以及拒访的情况，最终至少需要试验组86例，对照组86例，总计纳入样本量为172例。

四、病例选择

1. 入选标准

（1）病理证实胃癌；

（2）拟行胃癌根治术，围术期采用加速康复外科理念；

（3）年龄大于等于18岁。

2. 排除标准

（1）患者有良性前列腺增生病史、泌尿系统肿瘤病史、狭窄、畸形或手术史；

（2）使用影响排尿的药物；

（3）术前尿常规检查提示尿路感染。

3. 脱落标准

（1）手术过程中出现尿路损伤；

（2）出现危及生命的并发症；

（3）撤回知情同意。

五、研究方法与技术路线

1.导尿管管理

所有受试者在麻醉诱导后置入14F Foley导尿管，术后根据随机分组结果拔除导尿管。立即拔除导尿管组的患者在手术结束时直接在手术室拔除导尿管，<24小时拔除导尿管组的患者则在手术后24小时内拔除导尿管（ERAS指南的推荐治疗）。拔除导尿管后若出现明显尿意但无法排尿、下腹部疼痛等POUR症状则重新留置导尿管24小时。若拔除导尿管后6小时无法自主排尿但无POUR症状，则行膀胱B超，若膀胱残余尿量>800 mL则重新留置导尿管24小时。

2.其他围术期管理

患者围术期均采用加速康复外科管理方案，主要措施包括：（1）尽早拔除尿管、胃管、引流管等各种导管。（2）液体管理：术前不进行机械性肠道准备，术前6小时禁食、2小时禁饮，术前补充碳水化合物饮品。术中以维持体液平衡为目标，接受个体化的液体管理。患者清醒后开始少量饮水，不常规使用肠外营养；（3）镇痛管理：采用多模式镇痛方案，方案为“切口痛：罗哌卡因缝皮前局部浸润＋内脏痛：盐酸羟考酮口服＋炎性痛：氟比洛芬酯静滴”，患者根据自身情况酌情使用酒石酸布托啡诺喷鼻剂喷鼻。（4）活动管理：尽早停止心电监护，鼓励患者尽早下床活动。

六、观察项目与检测时点

1.主要结果：主要观察指标为术后急性尿潴留发病率。诊断标准为拔除导尿管后6小时无法自主排尿，出现排尿困难、腹胀腹痛等症状或膀胱B超显示膀胱残余尿量＞800mL。

2.次要结果：术后住院期间尿路感染发病率，诊断标准为排尿时有尿频、尿急、尿痛，或尿常规出现红细胞、白细胞、脓细胞等；舒适度：Kolcaba量表评价患者舒适度，分数越大表示患者舒适度越高，在术后第一天10:00评价；焦虑状态：HAD量表（hospital anxiety and depression scale）评估患者焦虑程度，分数≥11分表示存在焦虑状态，在术后第一天10:00评价。

3.其他结果：(1)一般资料：性别、年龄、ASA、BMI；(2)手术结果：手术类型、手术方式、手术时间，术中出血量，术中尿量，肿瘤分期；(3)恢复情况：记录术后住院天数，术后并发症（除POUR/UTI，根据Clavien-Dindo分级系统分级，III级及以上记录为严重并发症，术后首次排气时间，术后首次下床时间；(4)镇痛管理结果：记录术中、术后第0、1、2天阿片类药物消耗量，统一换算成吗啡毫克当量（POD 0 吗啡毫克当量指患者回到病房后至手术当日24:00）；术后第0、1、2天VAS评分；(5)液体管理结果：记录术中静脉补液量，术后静脉补液量。

七、不良事件

1.尿潴留：见干预措施部分。

2.其他不良事件可能包括导尿管相关性尿路感染、尿路损伤、膀胱痉挛、无法拔除导尿管等。导尿管评估至少每4小时进行一次。如果发生任何不良事件，将在病例报告表（CRF）中记录，并根据症状进行对症处理。根据需要拔除导尿管。

八、数据安全监查

本研究不存在重大安全性问题。我们认为数据监测委员会（DMC）的成本可能无法被其带来的益处所抵消。数据将由研究团队定期进行监测。

九、统计学处理

数据分析采用意向性分析（Intention-to-treat, ITT）方案。对于术后尿潴留（Postoperative Urinary Retention, POUR），采用非劣效性分析。如果立即拔除导尿管与早期拔除导尿管的POUR差异的双侧95%置信区间上限低于预先设定的10%的非劣效性界值，则认为立即拔除导尿管不劣于早期拔除导尿管。

分类变量以例数（百分比）表示，采用卡方检验（χ² test）或Fisher精确检验或Wilcoxon秩和检验进行比较，具体根据数据的分布情况而定。连续变量以均值（标准差）或中位数（四分位间距）表示，采用t检验或Mann-Whitney U检验进行分析，具体根据数据的正态性而定。所有统计分析均使用SPSS 26版（IBM公司）完成。所有统计检验均为双侧检验，P值小于0.05被认为具有统计学意义。

十、临床研究的伦理学

临床研究将遵循世界医学大会《赫尔辛基宣言》等相关规定。在研究开始之前，由伦理委员会批准该试验方案后才实施临床研究。每一位受试者入选本研究前，研究者有责任向受试者或其代理人完整、全面地介绍本研究的目的、程序和可能的风险，并签署书面知情同意书，应让受试者知道他们有权随时退出本研究，知情同意中应作为临床研究文件保留备查。研究过程中将保护受试者的个人隐私与数据机密性。
